# The economic value of identifying and treating Chagas disease patients earlier and the impact on *Trypanosoma cruzi* transmission

**DOI:** 10.1371/journal.pntd.0006809

**Published:** 2018-11-05

**Authors:** Sarah M. Bartsch, Cameron M. Avelis, Lindsey Asti, Daniel L. Hertenstein, Martial Ndeffo-Mbah, Alison Galvani, Bruce Y. Lee

**Affiliations:** 1 Public Health Computational and Operations Research, Johns Hopkins Bloomberg School of Public Health, Baltimore, MD, United States of America; 2 Global Obesity Prevention Center, Johns Hopkins Bloomberg School of Public Health, Baltimore, MD, United States of America; 3 Center for Infectious Disease Modeling and Analysis, Yale School of Public Health, New Haven, CT, United States of America; Sacro Cuore Hospital, ITALY

## Abstract

**Background:**

The World Health Organization’s 2020 Goals for Chagas disease include access to antiparasitic treatment and care of all infected/ill patients. Policy makers need to know the economic value of identifying and treating patients earlier. However, the economic value of earlier treatment to cure and prevent the Chagas’ spread remains unknown.

**Methods:**

We expanded our existing Chagas disease transmission model to include identification and treatment of Chagas disease patients. We linked this to a clinical and economic model that translated chronic Chagas disease cases into health and economic outcomes. We evaluated the impact and economic outcomes (costs, cost-effectiveness, cost-benefit) of identifying and treating different percentages of patients in the acute and indeterminate disease states in a 2,000-person village in Yucatan, Mexico.

**Results:**

In the absence of early treatment, 50 acute and 22 new chronic cases occurred over 50 years. Identifying and treating patients in the acute stage averted 0.5–5.4 acute cases, 0.6–5.5 chronic cases, and 0.6–10.8 disability-adjusted life years (DALYs), saving $694-$7,419 and $6,976-$79,950 from the third-party payer and societal perspectives, respectively. Treating in the indeterminate stage averted 2.2–4.9 acute cases, 6.1–12.8 chronic cases, and 11.7–31.1 DALYs, saving $7,666-$21,938 from the third-party payer perspective and $90,530-$243,068 from the societal perspective. Treating patients in both stages averted ≤9 acute cases and ≤15 chronic cases. Identifying and treating patients early was always economically dominant compared to no treatment. Identifying and treating patients earlier resulted in a cumulative cost-benefit of $7,273-$224,981 at the current cost of identification and treatment.

**Conclusions:**

Even when identifying and treating as little as 5% of cases annually, treating Chagas cases in the acute and indeterminate stages reduces transmission and provides economic and health benefits. This supports the need for improved diagnostics and access to safe and effective treatment.

## Author summary

The World Health Organization’s 2020 Goals for Chagas disease include access to antiparasitic treatment and care of all infected/ill patients. Policy makers need to know the economic value of identifying and treating patients earlier and what can be invested. We evaluated the impact and economic outcomes (costs, cost-effectiveness, cost-benefit) of identifying and treating different percentages of Chagas patients in the acute and indeterminate disease states using a transmission model linked to a clinical and economic outcomes model. Identifying and treating Chagas cases in the acute and indeterminate stages could result in up to an 18.0% relative reduction in transmission events as well as a 68.2% relative reduction in new chronic cases over 50 years compared to no treatment and was always economically dominant compared to no treatment. Identifying and treating Chagas disease in its earlier stages would reduce transmission and result in better health outcomes and cost-savings. In fact, the cost-savings would outweigh the cost of treating, meaning that earlier treatment may pay for itself. This supports the need for improved diagnostics and access to effective treatment.

## Introduction

While previous studies have evaluated the economic value of earlier treatment of Chagas disease (caused by the protozoan parasite *Trypanosoma cruzi*) in individuals, none have considered the spread or how it may prevent the spread of Chagas disease. Studies have estimated the cost of treating Chagas patients in various stages of disease[1, 2] and shown that the cost of Chagas disease is lower when treated in the acute stage[1]; however, these studies did not evaluate the cost-effectiveness of treatment nor did they consider the impact on the spread of *T*. *cruzi*. Another study has evaluated the cost-effectiveness (measured in cost per quality-adjusted life year gained) of vector control strategies plus drug treatment[3], but misses the broader effects of treatment (i.e., reduction in transmission). Treating patients in the earlier stages of infection can cure Chagas disease and is associated with a higher efficacy than in the chronic stage[4], which would lead to a reduction in each of the various forms of *T*. *cruzi* transmission (e.g., vectorial, congenital, and transfusional). Therefore, previous reports on the economic value of earlier treatment may underestimate the benefits of earlier treatment. While the World Health Organization’s (WHO) London Declaration 2020 Goals for Chagas disease cite “100% of countries with access to antiparasitic treatment” and “100% of infected/ill patients under care”[5], it is currently estimated that <1% of those infected with Chagas have access to care and treatment[6]. Given these 2020 goals, policy makers may want to know the economic value of identifying and treating patients earlier in the disease course to inform programs aimed at increasing access to care and treatment. Thus, the question remains, what is the economic value of earlier treatment of Chagas disease when considering transmission? Therefore, we modified our previously published *T*. *cruzi* transmission model[7] (calibrated to simulate a village in Yucatán, Mexico) and linked an economic outcomes model to evaluate the economic benefits (e.g., cost-effectiveness, cost-benefit) of treating Chagas cases in the acute and indeterminate stages.

## Methods

We adapted our previously published deterministic *T*. *cruzi* transmission model[7] in Python (Python Software Foundation, Wilmington, DE) by incorporating Chagas disease treatment, linked to a probabilistic clinical and economics model in Microsoft Excel (Microsoft, Redmond, WA) that utilized a Crystal Ball add-in (Oracle, Redwood City, CA) to translate chronic Chagas disease cases into health outcomes and corresponding costs and health effects. Our model represents a village in the Yucatán, Mexico. Mexico has one of the greatest burdens of Chagas disease worldwide (approximately 876,458 people are infected and 23.5 million are at risk for infection[8]) with 88% of the population potentially exposed to at least one competent vector species[9]. Chagas is endemic throughout the Yucatán peninsula, with 12–25 cases reported annually per 100,000 population over the last several years.[10, 11]

### Transmission model

**Fig 1:**
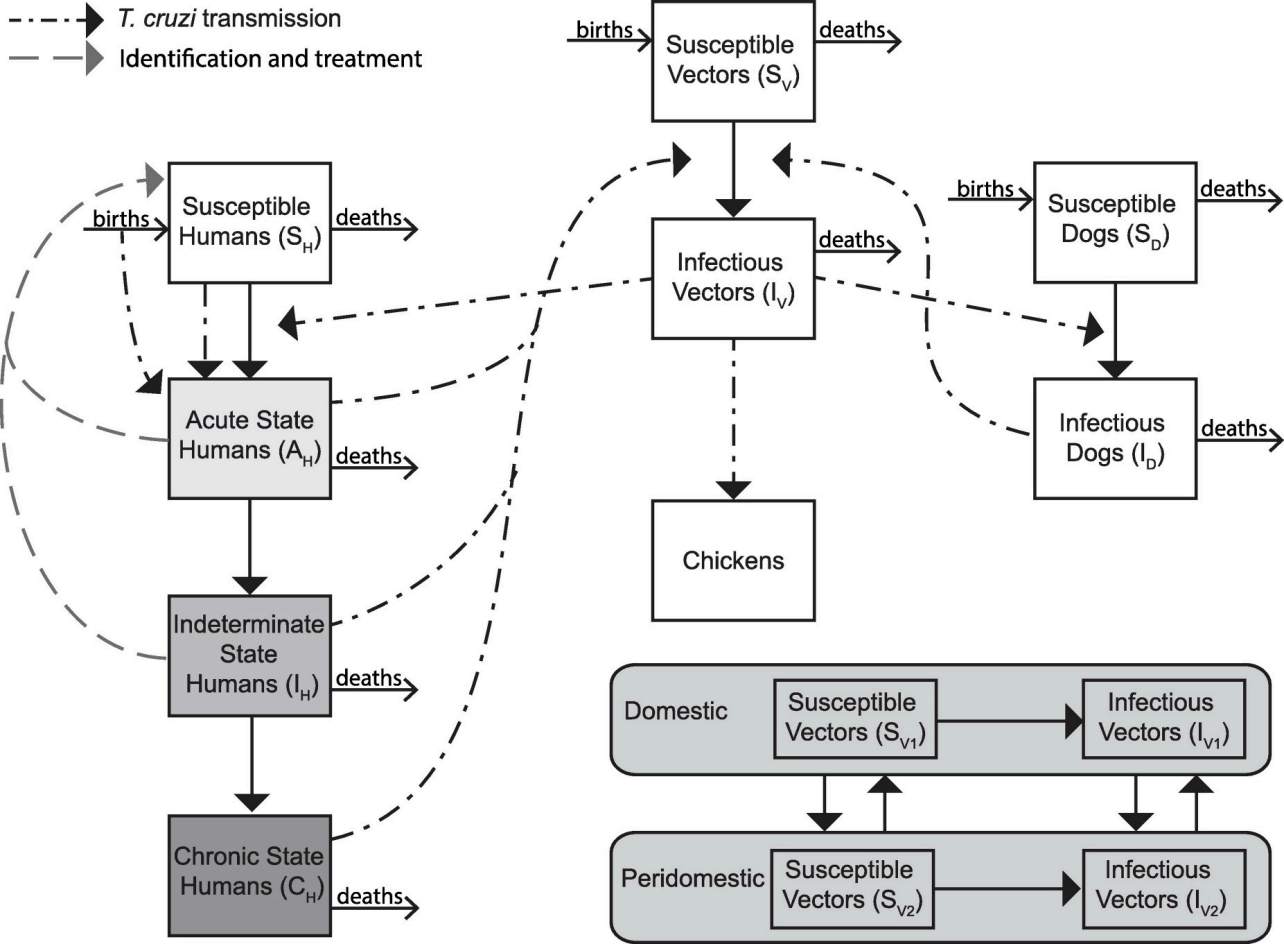
Transmission model structure. Deaths include Chagas related mortality in the acute and chronic states. Identification and treatment for states varies by scenario.

Our compartmental simulation model (outlined in Fig 1) includes vector-borne, congenital, and transfusional, forms of *T*. *cruzi* transmission. Briefly, it represents vector and host populations involved in *T*. *cruzi* transmission and included triatomines, human hosts, non-human hosts (i.e., dogs), and dead-end hosts (i.e., chickens) to simulate vector-borne transmission between these populations in both domestic and peridomestic settings, as well as congenital and transfusional transmission. During each time step (i.e., t = 1 month or 30 days; chosen as a balance between the shorter acute stage and longer indeterminate and chronic stages and to be consistent with other models), fixed fractions and rates determined the number of individuals in each compartment.

Each member of the human population could be in any of the following mutually exclusive disease states: susceptible (not infected with *T*. *cruzi* and able to become infected), acute Chagas disease (infected with *T*. *cruzi* and able to transmit, exhibiting mild and nonspecific symptoms, but in some cases can show Romaña’s sign or be serious and life-threatening, and having microscopically detectable parasitemia for 1.5 to 2 months), indeterminate Chagas disease (asymptomatically infected with *T*. *cruzi*, able to transmit), and chronic Chagas disease (infected with *T*. *cruzi*, able to transmit, and showing symptoms such as cardiomyopathy and/or megaviscera). Those not developing symptomatic chronic disease remained in the intermediate stage. Upon a feeding contact by an infectious triatomine, a susceptible human had a probability of becoming infected with *T*. *cruzi* via contamination with bug feces during or immediately after the feeding [represented in the vector-borne force of infection (FOI)]. Pregnant women had a probability of transmitting Chagas to their infants upon birth, with newborns becoming infected based on the congenital FOI. Additionally, a proportion of humans receiving a blood transfusion or organ transplant had a probability of becoming infected with *T*. *cruzi*, based on the transfusional FOI. Those in the acute and symptomatic chronic states of disease had probabilities of Chagas-related mortality. Triatomine bugs could be susceptible (not infected and able to become infected) or infectious (infected with *T*. *cruzi* and able to transmit to vertebrate hosts). Upon feeding on an infectious host, a susceptible bug had a probability of becoming infected with *T*. *cruzi*, conditional on the disease state of the host. Dogs served as reservoir hosts for *T*. *cruzi* and could be either susceptible or infected, with a susceptible dog becoming infected upon the bite of an infected vector based on the FOI. Dogs were considered competent transmitters and susceptible triatomines could become infected by an infected dog. Chickens served as dead end hosts, unable to become infected or further transmit *T*. *cruzi*.[12]

Our model included transmission in both domestic and peridomestic habitats, which vary by vector-host contact rates, and allowed for the movement of triatomines between habitats. Vectorial transmission was governed by the vectorial FOI. Consistent with other models of vector-borne diseases[13], this is a function of: (1) the triatomine biting rate, (2) the triatomine feeding proportion for each host type in each habitat, (3) the probability of transmission from vector to susceptible host, (4) the probability of transmission from infected host to susceptible bug, and (5) the proportion of infected hosts in each habitat. Transmission probabilities from vector to host varied with host species, while triatomine biting rates were assumed to be constant, regardless of host species.

During each time step, a proportion of the human population were identified and completed treatment with a standard course of benznidazole (5mg/kg/day for 60 days). Those treated had a probability of moving back to the susceptible state based on the efficacy of benznidazole, which was disease state-specific. Those unsuccessfully treated remain infected with *T*. *cruzi* and progress though the model. As antiparasitic treatment is only indicated once, those unsuccessfully treated were not eligible for additional antiparasitic treatment. In the absence of treatment, we assumed that once infected, persons were always infected.

### Economic model

Our economic model translated the number of cases identified and treated and number of new chronic cases generated from the transmission model into costs and health effects. The model included the cost associated with identification, diagnosis, treatment, treatment adverse events, and chronic disease costs and measured health effects in disability-adjusted life years (DALYs). Each individual completing treatment accrued the cost of identification, diagnosis, and treatment. Each individual also had a probability of adverse events and those who experienced adverse events accrued the cost of antihistamines, as dermatitis is the most common adverse event.[14] Case identification included the costs for overhead (e.g., materials, transport, telecommunications), personnel field work, which includes operational costs such as obtaining blood samples and patient data (estimated from personnel field time and hourly wage), and laboratory personnel for specimen processing, diagnostics and confirmation testing. Total diagnostic costs included a two-step process with a recombinant antigen enzyme-linked immunosorbent assay (ELISA) and chemiluminescense tests. Total treatment cost was determined from the dosage they received over the 60-day treatment course (i.e., cost per dose multiplied by dosage based on the individual’s weight).

Each chronic case from the transmission model accrued costs and health effects by multiplying each case by the annual cost and DALYs accrued per chronic case for the remainder of their lifetime. This was done by cumulating monthly cases to annual cases. For this cost and DALYs lost per case, we used our previously published Markov model, which provided the annual costs and DALYs accrued, specific to Latin America and discounted to net present value (NPV) with a 3% discount rate.[15] This model included costs and probabilities for diagnosis, treatment, and monitoring for chronic cases with and without cardiomyopathy and/or megaviscera, as well as procedures such as pacemaker implant and surgery for megacolon. DALYs were calculated as the years lost due to disability (YLD) and the years of life lost (YLL) as a result of Chagas-related mortality and included health-related outcomes such as cardiomyopathy, congestive heart failure, and megaviscera. The Markov model converted DALYs into productivity losses, thus representing indirect costs for the duration of disease and for early mortality.

Our economic model determined cost from the third-party payer and societal perspectives. The third-party payer perspective included direct costs (i.e., treatment and healthcare costs) and the societal perspective included direct plus indirect (i.e., productivity losses) costs.

For each scenario, we calculated both its cost-benefit and incremental cost-effectiveness ratio (ICER), as follows:

Cost‑Benefit=Benefit–Cost=DirectCostandProductivityLossesofAvertedChronicCases–CostofTreatment

$$\mathrm{ICER}=\frac{{\mathrm{Cost}}_{\mathrm{Earlier}\;\mathrm{Treatment}}{–\;\mathrm{Cost}}_{\mathrm{No}\;\mathrm{Earlier}\;\mathrm{Treatment}}}{{\mathrm{DALYs}}_{\mathrm{No}\;\mathrm{Earlier}\;\mathrm{Treatment}}{–\mathrm{DALYs}}_{\mathrm{Earlier}\;\mathrm{Treatment}}}$$

Results are in net present value (NPV), with all past and future costs and future DALYs discounted to 2018 $US using a 3% discount rate. ICERs were considered highly cost-effective if less than Mexico’s gross domestic product (GDP) per capita ($8,709[16]), cost-effective if 1 to 3 times the GDP, and not cost-effective if >3 times the GDP.

### Data inputs

Table 1 lists the model input parameters, values, and sources (with transmission model inputs adjusted to a monthly time step). The model is of a rural village (2,000 persons) in Yucatán, Mexico and in the absence of treatment, it was calibrated to assume a median *T*. *cruzi* prevalence value of 32.5% in *T*. *dimidiata*[17–25], and seroprevalence estimates of 1.85% in humans[25–35], and 14.58% in dogs[17, 28, 29, 36–39]. We assumed the impact of treatment in the chronic stage was negligible as it has limited effectiveness and takes years for sero-reversion to occur. As transmission probabilities and *T*. *dimidiata* feeding proportions across host species are highly variable and/or not well defined in the literature, these parameters were calibrated to available empirical data for the Yucatán. Efficacy and cost data were Mexico-specific when available and came from the scientific literature. Patient weight came from Mexico’s National Health and Nutrition survey.[40]

**Table 1:** Model input parameters, values, and sources.

Parameter	Value	Source
**Transmission Model**		
**Probabilities (% per month)**		
Developing chronic Chagas disease given indeterminate phase (% over 20 years)	25	[58]
Proportion time spent in domestic settings for humans (%)	90	Assumption
Proportion time spent in domestic settings for dogs (%)	23	[59]
Transmission to dogs given bite of infected vector (% per bite)	0.00274	Calibrated
Transmission to humans given bite of infected vector (% per bite)	0.00012	Calibrated
Transmission from acute stage to triatomine (% per bite)°	49.28	Calibrated
Transmission from indeterminate/chronic stage to triatomine (% per bite)°	1.62	Calibrated
Transmission from dog to triatomine (% per bite)°	19.00	Calibrated
Triatomine feeding proportion for humans in domestic settings	91.7	Calibrated
Triatomine feeding proportion for humans in peridomestic settings	46.7	Calibrated
Ratio of triatomine feeding proportion for chickens:dogs in peridomestic settings‡	51.7:48.3	Calibrated
Congenital transmission given birth from infected mother (% per birth)°	16.7	Calibrated
Relative prevalence of women of reproductive age as compared to the general population	93.3	Calibrated
Transmission via infected blood transfusion or organ transplant°	14.7	Calibrated
Human receiving a blood transfusion or organ donation	0.0042	Calibrated
Benznidazole treatment efficacy (per treatment)		
Acute stage	71.5	[60]
Indeterminate stage	57.6	[60]
**Rates (per month)**		
Triatomine biting rate	6	[61]
Triatomine birth rate (eggs hatching per month)^	32.13	[62]
Triatomine death rate*	0.036959468	[62, 63]
Triatomine in peridomestic habitat moves to domestic habitat	0.0205936	[64]
Triatomine in domestic habitat moves to peridomestic habitat	0.0205936	[64]
Human death rate^††^	0.001111111	[65]
Chagas related mortality during the acute stage	0.007178811	[66]
Movement from the acute phase to the indeterminate phase	0.04688	[58, 66]
Movement from the indeterminate phase to chronic phase	0.001198675	[58, 67]
Rate of Chagas-related mortality during chronic stage	0.00680	[68]
Dog death rate^††^	0.013888889	[69]
**Numbers**		
Number of humans	2,000	Assumption
Number of dogs	= N_H_/3.24	[59, 70]
Number of chickens†	= (N_H_/4)*0.5	[71]
Carrying capacity (per person)	50	Assumption
**Clinical and Economic Model**		
**Costs (2018 $US)**		
Hourly wage	6.82	[72]
Case identification overhead	1.92	[50]
Diagnostic tests	10.80	[73]
Case identification laboratory work	1.81	[50]
Benznidazole (100mg)	0.14–0.33	[74]
Adverse events (antihistamines)	2.19 (0.77–5.25)	[75]
Chronic Chagas case (annual healthcare, Lee)	457 (247–759)	[15]
Chronic Chagas case (annual societal, Lee)	4,847 (4,262–5,294)	[15]
Chronic Chagas case, undiagnosed (annual societal, Ramsey)	19,857 (17,871–21,843)	[1]
Chronic Chagas case, diagnosed (annual societal, Ramsey)	29,359 (26,423–32,296)	[1]
**Probabilities (%)**		
Interrupt treatment/adverse events	11.25 (1.06)	[76, 77]
**Disability-adjusted life years (DALYs)**		
Chronic Chagas disease (annual)	0.51 (0.38–0.6)	[15]
**Numbers**		
Case identification personnel hours	1.54	[50]
Patient weight (kg)	52.1 (2–188)	[40]
Treatment duration (days)	60	

°Calibrated values fall within range of limited published literature [73, 78–81]

‡Calibrated values for remainder of feeding proportion after humans, where chicken proportion is higher than for dogs following the literature[81, 82]

^Assumes maximum number of eggs laid per day and egg hatching rate (90%) from literature[62] and assumes 7% of vector population are adult females

*Calculated using the adult life span and days from egg to adult to determine total life span

††Model assumes constant populations, such that human birth rate is equal to natural death rate and Chagas death rate and dog birth rate is same as death rate

†Assumes 50% of households have chickens and that there are 4 to 5 persons per household

#### Scenarios and sensitivity analyses

Our baseline scenario assumed routine treatment of chronic cases (i.e., no early treatment), which did not impact baseline prevalence, while experimental scenarios implemented identification and treatment in the acute phase, in the indeterminate phase, and in both the acute and indeterminate phase.

Each experiment consisted of running the transmission model for 50 years, while the economic model utilized Monte Carlo simulations of 10,000 trials, varying each parameter across their ranges. Sensitivity analyses varied the percentage of the population being identified and completing treatment each year (5% to 100%) and the cost of benznidazole ($0.14 to $1.50 per 100mg pill). Additional scenarios utilized the annual cost per chronic case for Mexico reported by Ramsey et al (Table 1).[1] We also varied the total cost of treatment per person ($50 to $300) in the cost-benefit analysis. Results are presented as median and 95% uncertainty interval (95% UI).

## Results

### No early treatment (Routine chronic treatment)

**Fig 2:**
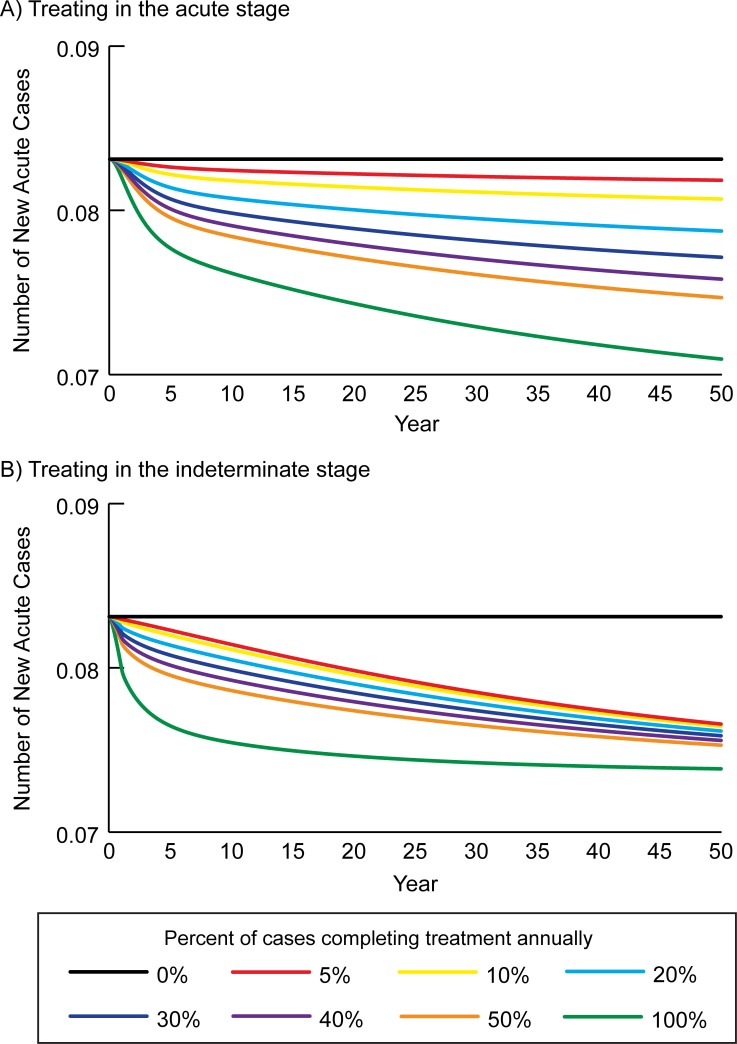
Number of new acute cases over time when identifying and treating different percentages of Chagas patients annually in the A) acute stage, and B) the indeterminate stage.

In the absence of treatment in the acute and indeterminate stages, Chagas prevalence was maintained at 1.8% in humans, with 50 new acute cases (i.e., transmission events) and 22 new chronic cases over 50 years; Fig 2 shows the number of new acute cases over time. Table 2 shows the number of new chronic cases and annual costs and DALYs accrued over time. Overall, chronic cases accrued a total lifetime cost of $44,955 (95% UI: $27,856–65,574) from the third-party payer perspective, $444,483 (95% UI: $404,803–479,798) from the societal perspective, and accrued 55.8 (95% UI: 45.2–64.5) DALYs. This translates to $450 and $4,444 per 1,000 person-years from the third-party payer and societal perspectives, respectively.

**Table 2:** Cumulative number of acute Chagas cases identified and completing treatment and the impact of treating in Chagas disease in the acute stage on the number of chronic cases and median cost (95% uncertainty interval) over time when identifying and treating different percentages of patients annually.

Percentage of Cases Identified and Completing Treatment Annually	Number Identified and Completing Treatment	Number New Chronic Cases	Third-Party Payer Costs	Societal Costs	DALYs
**Year 1**					
No Treatment	-	0.4	202 (125–294)	2,025 (1,844–2,185)	0.3 (0.2–0.3)
5%	0.1	0.4	206 (131–302)	2,035 (1,856–2,190)	0.3 (0.2–0.3)
20%	0.3	0.4	221 (146–318)	2,046 (1,864–2,200)	0.3 (0.2–0.3)
50%	0.7	0.4	250 (168–350)	2,071 (1,878–2,225)	0.3 (0.2–0.3)
100%	1.2	0.4	284 (188–390)	2,111 (1,925–2,286)	0.3 (0.2–0.3)
**Year 5**					
No Treatment	-	2.2	2,487 (1,541–3,628)	24,959 (22,730–26,942)	3.1 (2.5–3.6)
5%	0.4	2.2	2,508 (1,578–3,686)	25,038 (22,829–26,936)	3.1 (2.5–3.6)
20%	1.3	2.2	2,573 (1,656–3,732)	24,992 (22,768–26,895)	3.1 (2.5–3.6)
50%	2.7	2.2	2,682 (1,773–3,790)	25,000 (22,736–26,910)	3.1 (2.5–3.6)
100%	4.2	2.1	2,722 (1,774–3,851)	25,088 (22,854–27,007)	3.1 (2.5–3.5)
**Year 10**					
No Treatment	-	4.4	7,243 (4,488–10,565)	72,679 (66,190–78,455)	9.0 (7.3–10.4)
5%	0.7	4.4	7,271 (4,556–10,689)	72,752 (66,337–78,264)	9.0 (7.4–10.4)
20%	2.5	4.3	7,367 (4,686–10,713)	72,189 (65,807–77,735)	9.0 (7.4–10.4)
50%	5.0	4.2	7,519 (4,907–10,664)	71,578 (65,099–77,033)	8.9 (7.2–10.2)
100%	7.5	4.1	7,475 (4,831–10,668)	71,157 (64,794–76,622)	8.7 (7.1–10.1)
**Year 20**					
No Treatment	-	8.8	18,237 (11,301–26,602)	182,994 (166,655–197,536)	22.6 (18.3–26.2)
5%	1.4	8.7	18,199 (11,379–26,759)	182,334 (166,253–196,145)	22.6 (18.4–26.1)
20%	4.9	8.4	18,136 (11,507–26,444)	178,817 (163,021–192,555)	22.2 (18.3–25.7)
50%	9.6	8.1	18,071 (11,754–25,779)	174,460 (158,642–187,744)	21.6 (17.5–24.9)
100%	14.1	7.8	17,643 (11,342–25,267)	170,603 (155,348–183,748)	20.9 (17.0–24.3)
**Year 30**					
No Treatment	-	13.2	28,025 (17,366–40,880)	281,211 (256,102–303,558)	34.8 (28.1–40.2)
5%	2.1	13.0	27,832 (17,393–40,931)	279,005 (254,397–300,135)	34.7 (28.2–40.0)
20%	7.3	12.4	27,434 (17,330–39,994)	270,718 (246,808–291,519)	33.6 (27.7–38.9)
50%	14.1	11.7	26,862 (17,412–38,373)	260,259 (236,682–280,090)	32.2 (26.1–37.2)
100%	20.5	11.0	25,796 (16,550–37,039)	250,809 (228,380–270,132)	30.8 (25.0–35.7)
**Year 40**					
No Treatment	-	17.6	35,770 (22,165–52,176)	358,922 (326,874–387,444)	44.4 (35.9–51.3)
5%	2.8	17.2	35,388 (22,108–52,044)	354,810 (323,515–381,680)	44.1 (35.9–50.8)
20%	9.7	16.3	34,558 (21,815–50,381)	341,143 (311,017–367,357)	42.3 (34.9–49.0)
50%	18.6	15.0	33,356 (21,603–47,729)	323,803 (294,494–348,500)	40.1 (32.5–46.2)
100%	26.8	13.8	31,639 (20,284–45,443)	308,119 (280,561–331,847)	37.8 (30.8–43.9)
**Year 50**					
No Treatment	-	22.0	41,665 (25,818–60,775)	418,074 (380,745–451,297)	51.7 (41.8–59.7)
5%	3.5	21.4	41,093 (25,668–60,434)	412,046 (375,704–443,253)	51.2 (41.7–59.0)
20%	12.0	20.0	39,825 (25,133–58,059)	393,208 (358,486–423,424)	48.8 (40.2–56.5)
50%	23.0	18.2	38,005 (24,605–54,382)	369,285 (335,874–397,466)	45.7 (37.0–52.7)
100%	33.1	16.5	35,677 (22,866–51,251)	347,670 (316,574–374,439)	42.7 (34.7–49.6)

DALYs = disability-adjusted life years

Using the cost per case from Ramsey et al., societal costs totaled $1.8 million (95% UI: $1.6–1.9 million) assuming undiagnosed Chagas disease and $2.7 million (95% UI: $2.5–2.9 million) assuming all chronic cases are diagnosed and treated.

### Identifying and treating patients in the acute stage

Fig 2A shows how treating Chagas in the acute stage impacts transmission over time, with treatment averting 0.5 to 5.4 new acute cases per 2,000 population over 50 years (1.0–10.8% relative reduction compared to no treatment), when 5% to 100% of cases detected and completed treatment annually. Table 2 shows the clinical and economic outcomes over time when different percentages of acute stage cases were identified and completed treatment annually. While all scenarios accrued costs in year 1, cost-savings began to manifest by year 10. Identifying and treating Chagas in the acute stage resulted in a 2.7–25.0% relative reduction in new chronic cases (0.6 to 5.5 new cases) over 50 years (Table 2). Identifying and treating in the acute stage (5% to 100% of cases annually) resulted in cost-savings totaling $694 to $7,419 from the third-party payer perspective, $6,976 to $79,950 from the societal perspective, and averting 0.6 to 10.8 DALYs over the lifetime of all chronic cases occurring over the 50-year period. These NPV lifetime costs were $375 to $443 per 1,000 person-years from the third-party payer perspective and $3,645 to $4,374 per 1,000 person-years from the societal perspective.

Assuming the cost per case from Ramsey et al., total societal costs ranged from $1.5 to $1.8 million (if chronic cases remain undiagnosed) to $2.2 to $2.6 million (assuming all chronic cases are diagnosed and treated), varying by the proportion (5% to 100%) of acute cases identified and completing treatment annually. Total cost-savings ranged from $56,171 to $1.3 million, depending on the cost of the chronic case and the percent treated annually.

**Fig 3:**
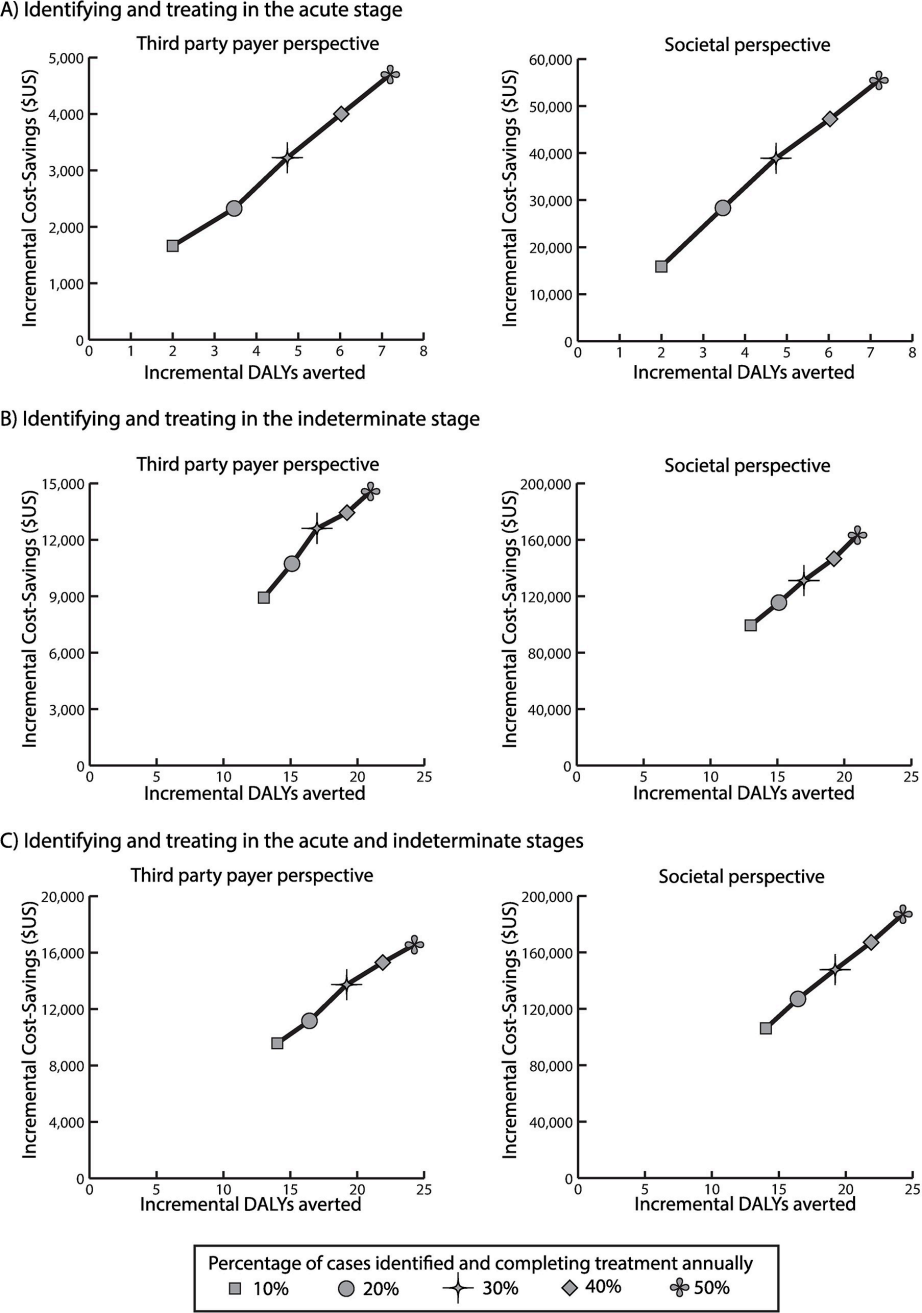
Incremental cost and effectiveness of compared to no earlier treatment from the third-party payer and societal perspectives when identifying and treating an increasing proportion of Chagas patients in A) the acute stage, B) the indeterminate stage, and C) the acute and indeterminate stages. Costs and disability-adjusted life years (DALYs) are in net present value (2018 $US).

**Fig 4:**
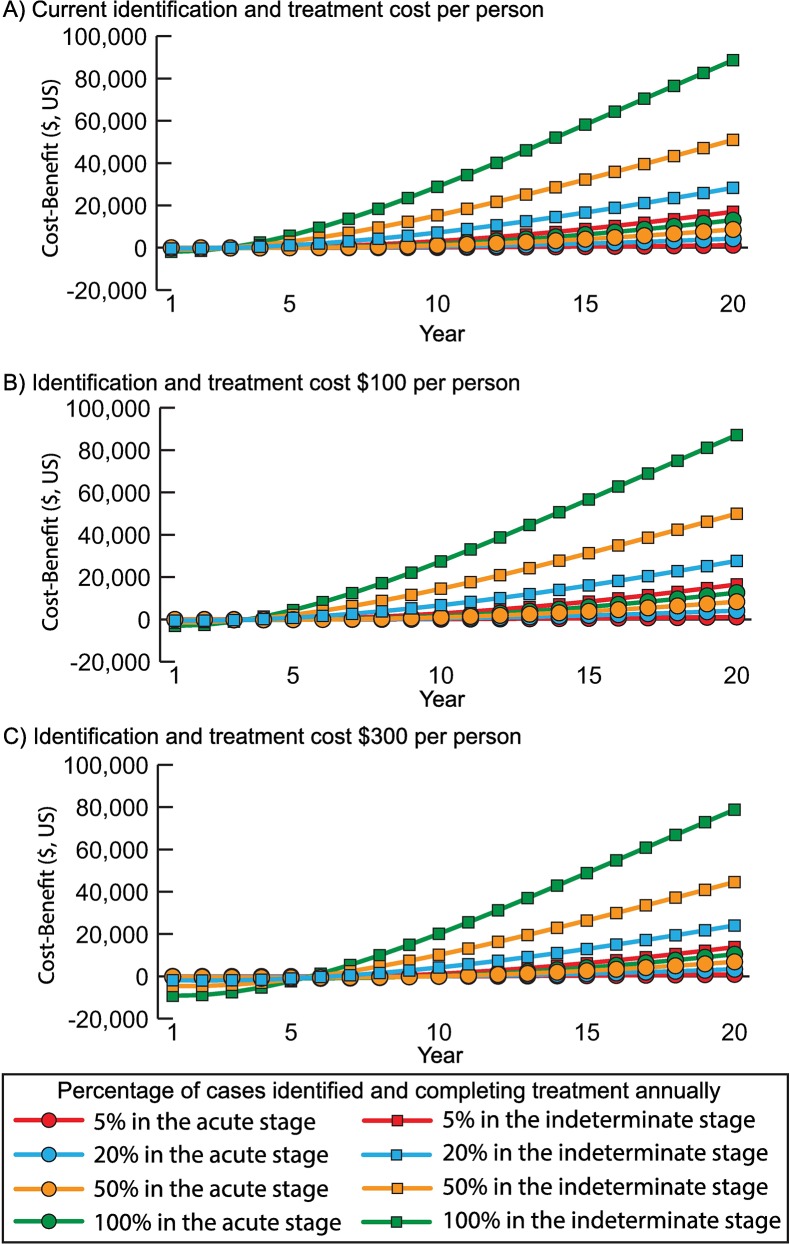
Cost-benefit (net present value, 2018 $US) over time of when identifying and treating different percentages of Chagas patients in the acute or indeterminate stage annually compared to no treatment from the societal perspective when treatment cost A) $50 per person, B) $100 per person, and C) $300 per person.

Identifying and treating Chagas disease in the acute stage was economically dominant (i.e., saved costs and provided health benefits) compared to no treatment from both perspectives. At $1.50 per 100mg of benznidazole, treating 5% of cases was cost-effective (ICER $361/DALY averted) and dominant when treating ≥10% of cases. Fig 3A shows the incremental cost and effectiveness of increasing the proportion of acute cases treated compared to baseline. Fig 4 shows the cumulative cost-benefit of earlier treatment over time for various treatment costs. At the current cost (~$63, varying by weight), identifying and treating Chagas patients in the acute stage would generate a positive return by year 6, resulting in a cumulative cost-benefit of $7,273 to $71,705 over 50 years (treating 5% to 100% of acute patients annually; Fig 4A). With a $300 cost, positive returns started in year 10 (Fig 4C).

### Identifying and treating patients in the indeterminate stage

Identifying and treating Chagas disease in the indeterminate stage averted 2.2 to 4.9 new acute cases (4.4–9.8% relative reduction) per 2,000 population (5% to 100% of cases were detected and completed treatment annually) over 50 years (Fig 2B). Identifying and treating in the indeterminate stage, 41.3 to 68.1 cases were treated (treating 5% to 100% annually), averting 6.1 to 12.8 new chronic cases (27.7–58.2% relative reduction) over 50 years (Table 3). Over the 50-year period, cost-savings totaled $7,666 to $21,938 from the third-party payer perspective, $90,530 to $243,068 from the societal perspective, while 11.7 to 31.1 DALYs were averted. These NPV lifetime costs were $230 to $372 and $2,013 to $3,539 per 1,000 person-years from the third-party payer and societal perspectives, respectively.

**Table 3:** Cumulative number of indeterminate Chagas cases identified and completing treatment and the impact of treating in Chagas disease in the indeterminate stage on the number of chronic cases and median cost (95% uncertainty interval) over time when identifying and treating different percentages of patients annually.

Percentage of Cases Identified and Completing Treatment Annually	Number Identified and Completing Treatment	Number New Chronic Cases	Third-Party Payer Costs	Societal Costs	DALYs
**Year 1**					
No Treatment	-	0.4	202 (125–294)	2,025 (1,844–2,185)	0.3 (0.2–0.3)
5%	1.5	0.4	307 (203–420)	2,129 (1,945–2,313)	0.3 (0.2–0.3)
20%	6.1	0.4	581 (382–952)	2,399 (2,121–2,797)	0.3 (0.2–0.3)
50%	15.3	0.4	1,143 (701–2,151)	2,973 (2,487–3,969)	0.3 (0.2–0.3)
100%	30.6	0.3	2,080 (1,204–3,862)	3,896 (2,983–5,715)	0.3 (0.2–0.3)
**Year 5**					
No Treatment	-	2.2	2,487 (1,541–3,628)	24,959 (22,730–26,942)	3.1 (2.5–3.6)
5%	4.9	2.1	2,780 (1,794–3,879)	24,776 (22,629–26,656)	3.0 (2.4–3.5)
20%	9.5	1.9	2,917 (1,978–4,070)	23,845 (21,676–25,678)	2.9 (2.4–3.4)
50%	18.6	1.6	3,298 (2,264–4,709)	22,185 (20,098–24,025)	2.6 (2.1–3.0)
100%	33.8	1.1	3,822 (2,557–5,982)	19,328 (17,311–21,579)	2.1 (1.8–2.5)
**Year 10**					
No Treatment	-	4.4	7,243 (4,488–10,565)	72,679 (66,190–78,455)	9.0 (7.3–10.4)
5%	9.1	4.0	7,541 (4,802–10,591)	69,835 (63,674–75,127)	8.6 (6.9–9.9)
20%	13.6	3.7	7,225 (4,787–10,353)	65,514 (59,653–70,511)	8.0 (6.6–9.3)
50%	22.7	3.1	7,109 (4,814–9,681)	57,492 (52,268–61,985)	7.0 (5.7–8.0)
100%	37.7	2.0	6,498 (4,438–9,292)	43,943 (39,908–47,601)	5.2 (4.2–6.0)
**Year 20**					
No Treatment	-	8.8	18,237 (11,301–26,602)	182,994 (166,655–197,536)	22.6 (18.3–26.2)
5%	17.3	7.5	17,676 (11,140–24,787)	166,545 (151,877–179,170)	20.6 (16.6–23.7)
20%	21.8	7.0	16,351 (10,649–23,654)	154,593 (140,740–166,705)	19.0 (15.6–22.1)
50%	30.6	5.8	14,890 (9,821–20,609)	132,041 (119,911–142,312)	16.2 (13.2–18.6)
100%	45.4	3.9	11,814 (8,024–16,542)	94,478 (86,043–101,689)	11.4 (9.4–13.2)
**Year 30**					
No Treatment	-	13.2	28,025 (17,366–40,880)	281,211 (256,102–303,558)	34.8 (28.1–40.2)
5%	25.4	10.6	25,780 (16,260–36,191)	244,252 (222,750–262,810)	30.3 (24.3–34.8)
20%	29.8	9.8	23,662 (15,307–34,413)	226,372 (206,089–244,156)	27.9 (22.8–32.4)
50%	38.5	8.3	21,251 (13,919–29,486)	192,722 (175,165–207,715)	23.8 (19.4–27.3)
100%	53.0	5.7	16,246 (10,932–22,834)	136,705 (124,473–147,208)	16.6 (13.6–19.2)
**Year 40**					
No Treatment	-	17.6	35,770 (22,165–52,176)	358,922 (326,874–387,444)	44.4 (35.9–51.3)
5%	33.4	13.4	31,552 (19,888–44,350)	299,628 (273,247–322,424)	37.2 (29.8–42.7)
20%	37.7	12.5	28,951 (18,644–42,173)	277,888 (252,976–299,787)	34.2 (28.0–39.9)
50%	46.3	10.6	25,816 (16,922–35,893)	237,061 (215,422–255,510)	29.3 (23.9–33.6)
100%	60.5	7.5	19,643 (13,129–27,742)	169,008 (153,802–182,034)	20.6 (16.9–23.8)
**Year 50**					
No Treatment	-	22.0	41,665 (25,818–60,775)	418,074 (380,745–451,297)	51.7 (41.8–59.7)
5%	41.3	15.9	35,546 (22,393–49,973)	337,743 (308,016–363,465)	41.9 (33.6–48.2)
20%	45.5	14.9	32,615 (20,969–47,564)	313,663 (285,522–338,370)	38.6 (31.7–45.0)
50%	54.0	12.7	29,102 (19,036–40,573)	268,465 (243,964–289,344)	33.2 (27.0–38.1)
100%	68.1	9.2	22,186 (14,803–31,396)	193,055 (175,765–207,928)	23.6 (19.4–27.2)

DALYs = disability-adjusted life years

Assuming the cost of an undiagnosed chronic case, early treatment in the indeterminate stage cost $0.8 to $1.4 million (saving $1.3 to $1.9 million), while it cost $1.2 to $2.1 million assuming chronic cases are treated (saving $0.6 to $1.5 million) from the societal perspective.

Identifying and treating in the indeterminate stage was economically dominant compared to no treatment for all strategies tested from both perspectives, even at a cost of $1.50 per 100mg. It was also more cost-effective than treating in the acute stage, so that treating 5% of indeterminate patients annually was dominant compared to identifying and treating 100% of acute patients. Fig 3B shows the incremental gains of increasing the proportion treated. At the current treatment cost (Fig 4A), positive returns were seen by year 3 (50%-100% treated) or year 4 (5%-20% treated), resulting in a total cost-benefit of $81,352 to $224,981 over 50 years. At $300 per person (Fig 4C), positive returns started between year 6 (treating 100% annually) and year 8 (treating 5% annually).

### Identifying and treating patients in the acute and indeterminate stages

Identifying and treating Chagas patients in the acute and indeterminate stages averted 2.7 to 9.0 new acute cases (5.4–18.0% relative reduction) per 2,000 persons over 50 years. Table 4 shows the clinical and economic outcomes over time. Scenarios garnered cost-savings by year 5 (Table 4). Identifying and treating in both stages averted 6.3 to 15.0 new chronic cases (28.6–68.2% relative reduction) over the 50-year period, saving a total NPV of $8,420 to $25,076 from the third-party payer perspective, $94,989 to $279,379 from the societal perspective, and 12.3 to 35.7 DALYs over the lifetime of all chronic cases that occurred during the 50-year period. The NPV lifetime costs per 1,000 person-years were $199 to $365 from the third-party payer perspective and $1,650 to $3,494 from the societal perspective.

**Table 4:** Cumulative number of acute and indeterminate Chagas cases identified and completing treatment and the impact of treating in Chagas disease in the acute and indeterminate stages on the number of chronic cases and median cost (95% uncertainty interval) over time when identifying and treating different percentages of patients annually.

Percentage of Cases Identified and Completing Treatment Annually	Number Identified and Completing Treatment	Number New Chronic Cases	Third-Party Payer Costs	Societal Costs	DALYs
**Year 1**					
No Treatment	-	0.4	202 (125–294)	2,025 (1,844–2,185)	0.3 (0.2–0.3)
5%	1.6	0.4	310 (208–429)	2,130 (1,945–2,308)	0.3 (0.2–0.3)
20%	6.4	0.4	604 (393–998)	2,424 (2,150–2,853)	0.3 (0.2–0.3)
50%	16.0	0.4	1,195 (703–2,159)	3,010 (2,486–4,018)	0.3 (0.2–0.3)
100%	31.8	0.3	2,138 (1,223–4,168)	3,986 (2,998–6,004)	0.3 (0.2–0.3)
**Year 5**					
No Treatment	-	2.2	2,487 (1,541–3,628)	24,959 (22,730–26,942)	3.1 (2.5–3.6)
5%	5.1	2.1	2,772 (1,839–3,867)	24,691 (22,580–26,597)	3.0 (2.5–3.5)
20%	10.2	1.9	3,018 (1,998–4,148)	23,873 (21,844–25,849)	2.9 (2.3–3.3)
50%	20.2	1.6	3,390 (2,302–4,812)	22,267 (20,118–24,250)	2.6 (2.1–3.0)
100%	36.2	1.1	3,942 (2,568–6,273)	19,284 (17,234–21,853)	2.1 (1.7–2.5)
**Year 10**					
No Treatment	-	4.4	7,243 (4,488–10,565)	72,679 (66,190–78,455)	9.0 (7.3–10.4)
5%	9.4	4.0	7,481 (4,894–10,555)	69,530 (63,548–74,882)	8.6 (7.0–9.9)
20%	14.8	3.7	7,431 (4,933–10,414)	65,249 (59,546–70,574)	8.0 (6.5–9.3)
50%	24.9	3.0	7,178 (4,873–9,890)	56,967 (51,731–61,478)	6.8 (5.6–7.9)
100%	41.0	1.9	6,624 (4,266–9,504)	42,872 (39,141–46,910)	5.0 (4.1–5.8)
**Year 20**					
No Treatment	-	8.8	18,237 (11,301–26,602)	182,994 (166,655–197,536)	22.6 (18.3–26.2)
5%	17.9	7.5	17,463 (11,307–24,768)	165,509 (151,252–178,296)	20.5 (16.6–23.6)
20%	23.6	6.8	16,610 (10,781–23,565)	152,698 (139,249–165,269)	18.9 (15.3–21.8)
50%	34.1	5.5	14,751 (9,960–20,332)	128,475 (116,747–138,396)	15.6 (12.7–18.0)
100%	50.4	3.4	11,577 (7,582–15,962)	88,451 (81,150–95,751)	10.6 (8.7–12.3)
**Year 30**					
No Treatment	-	13.2	28,025 (17,366–40,880)	281,211 (256,102–303,558)	34.8 (28.1–40.2)
5%	26.2	10.5	25,395 (16,453–36,078)	242,169 (221,342–260,936)	30.1 (24.4–34.5)
20%	32.2	9.5	23,830 (15,385–34,007)	221,931 (202,286–240,218)	27.5 (22.2–31.7)
50%	43.1	7.7	20,607 (13,806–28,544)	184,083 (167,355–198,326)	22.5 (18.3–25.9)
100%	59.5	4.8	15,317 (9,981–21,042)	123,212 (113,218–133,257)	14.9 (12.2–17.3)
**Year 40**					
No Treatment	-	17.6	35,770 (22,165–52,176)	358,922 (326,874–387,444)	44.4 (35.9–51.3)
5%	34.3	13.2	31,015 (20,104–44,093)	296,507 (270,985–319,480)	36.8 (29.9–42.3)
20%	40.7	11.9	28,915 (18,629–41,316)	270,549 (246,520–292,862)	33.5 (27.1–38.6)
50%	52.0	9.5	24,745 (16,550–34,277)	222,746 (202,580–239,989)	27.2 (22.2–31.4)
100%	68.6	6.0	17,960 (11,634–24,531)	147,414 (135,525–159,329)	17.9 (14.6–20.7)
**Year 50**					
No Treatment	-	22.0	41,665 (25,818–60,775)	418,074 (380,745–451,297)	51.7 (41.8–59.7)
5%	42.3	15.7	34,874 (22,607–49,576)	333,688 (304,954–359,541)	41.4 (33.6–47.6)
20%	49.1	14.1	32,386 (20,838–46,295)	303,506 (276,540–328,573)	37.6 (30.4–43.4)
50%	60.7	11.2	27,515 (18,381–38,148)	248,808 (226,286–268,023)	30.4 (24.8–35.0)
100%	77.5	7.0	19,756 (12,730–26,997)	163,830 (150,721–177,182)	19.9 (16.3–23.1)

DALYs = disability-adjusted life years

Earlier treatment in both stages garnered societal cost-savings up to $2.1 million (undiagnosed chronic case cost and 100% early treatment annually) or $1.7 million (100% chronic diagnosed and treated and 100% early treatment annually).

Identifying and treating in both stages (≥5% annually) was dominant compared to no treatment and compared to treating 100% of acute patients and a similar percent of indeterminate patients. The incremental gains were largest when treating both acute and indeterminate patients earlier (Fig 3C). At the current cost per person, treatment yielded a positive return by year 3, except when treating only 5% of cases annually (positive by year 4), with a total return of $84,390 to $253,850 by year 50. At $100 per person, positive returns took 4 to 5 years to manifest, totaling up to $251,797 over 50 years; while at $300 per person, positive returns were seen between years 6 and 9, totaling up to $240,581 over 50 years.

## Discussion

Identifying and treating Chagas disease in its earlier stages (i.e., acute and indeterminate) would result in reduced transmission, better health outcomes, and cost-savings. Treatment in either stage could save up to $279,379 or $2.6 million (varying with the cost of a chronic Chagas case) and 35.7 DALYs in a 2,000-peron village in the Yucatán. In fact, the cost-savings would outweigh the cost of identifying and treating earlier, meaning that earlier treatment may pay for itself. While treatment in the acute stage provided a greater reduction in transmission (as it has a higher transmission rate than the later disease stages), treatment in the indeterminate stage provided a greater reduction in the number of new chronic cases (given a larger proportion of the population is in the indeterminate stage, following Chagas natural disease course). Thus, treatment in the indeterminate stage would cover more of the population in the first year. Overall, earlier treatment resulted in a 9% relative reduction in transmission events over 5 years which is substantially less than the 83% reduction garnered by reducing domestic vectorial, congenital, and transfusional transmission[7]; however, this reduction is not inconsequential as it leads to cost-savings.

To put this in perspective, vector control programs in Latin American and the Caribbean are dominant compared to no control programs, while a vector control program plus drug treatment was cost-effective compared to treatment control alone (ICER: $699/quality-adjusted life year in 2002 $US).[3] Various insecticide spraying strategies in Northwestern Argentina cost $83 to $205 per case averted (in 2004 $US).[41] In Mexico, various control measures are estimated to be effective and cost a total of $7–8 per house for insecticide spraying and peri-domicile cleaning, while insect screens and impregnated curtains are estimated to cost $55–69 per house, while impregnating currently existing curtains cost $3–4 per house (total costs include materials and labor for each, discounted to 2018 $US).[42, 43] In Bolivia, an effective program to control congenital transmission by detecting and treating all cases would require an investment of $123 per infected newborn ($1.2 per newborn).[44]

Our results give an idea of what cost-savings could be provided by earlier treatment and thus, what could be spent on strategies to achieve this and into reaching the treatment- and care-related WHO London Declaration of 2020 goals. However, given that <1% of those infected with Chagas have access to care and treatment[6], great strides should be taken to expand access and provide adequate diagnostics and affordable treatment. Evidence suggests there are several obstacles to access of Chagas treatment in Mexico, which include regulatory, financing, and organizational challenges.[45] For example, benznidazole is excluded from the national formulary (but has a commercial permit by Cofepris as of mid-2017[46]), there are no national guidelines for Chagas disease treatment, and there is insufficient training and education of providers about Chagas.

Most Chagas disease prevention and control seems to focus on vector control, however, our results show there is value in earlier identification and treatment of infections. Thus, policy makers should advocate for earlier treatment and our results can help inform spending into securing Chagas chemotherapy. Third-party payers and government healthcare systems should advocate for earlier treatment to reduce the subsequent healthcare burden they may later incur by providing for chronic Chagas cases. Additionally, clinicians and healthcare providers should be trained to better identify Chagas and to provide appropriate treatment. People should be educated on the signs and symptoms of *T*. *cruzi* infection and should be encouraged to seek care. This may require the use of behavior and/or Chagas awareness campaigns, which could highlight and promote the importance of adequate diagnostics and available treatment. In the Yucatán peninsula, while many people are knowledgeable of triatomines and their habits, most had a limited understanding of transmission and clinical outcomes of Chagas disease.[47] Raising this awareness could lead to increases in diagnosis and treatment and a subsequent reduction in the incidence and prevalence of Chagas in the region.

It should be noted that there is wide debate over cost-effectiveness thresholds[48, 49]. Other thresholds proposed are opportunity-cost based (i.e., the cost of generating health in the present health system) which can inform resource allocation decisions and suggest that the routinely used GDP thresholds may be too high.[48] Thus, care should be taken to consider local circumstances and context when interpreting our results. Although our study focuses on Chagas in the Yucatán peninsula, our results can be used in the context of other regions. Earlier treatment may provide similar benefits in regions with comparable prevalence, for example, other states in Mexico (e.g., Puebla and Durango[30]) and other endemic areas (e.g., urban Arequipa, Peru[50], Ceará State and Rondônia State in Brazil[51]). Additionally, earlier treatment may be more effective in a places with a higher prevalence (e.g., Querétaro, Mexico[30], Gran Chaco region[52–54], rural Lara State and Barinas State in Venezuela[55, 56]). Earlier treatment may also result in similar benefits in regions with a similar annual cost per case [e.g., $1,555 (in 2018 $US) in Colombia[2]]. However, our results may not readily extend to regions with a lower prevalence, different standard of care, or those where vector-borne transmission has been interrupted or is rare (e.g., Chile, Uruguay, and non-endemic countries).

### Limitations

All models are simplifications of real life and as such cannot represent every possible event or outcome. Our current model is deterministic in nature and does not include the full heterogeneity possible for Chagas disease transitions between states. Our model inputs were fit to disparate data of varying quality yet can be refined as new data become available. As Chagas disease is underdiagnosed and underreported, our estimates for *T*. *cruzi* seroprevalence are subject to limitations; however, we used the best available data for these parameters. Our results only account for those patients completing treatment; thus, our 100% completing treatment scenarios optimistically assume that no one discontinues treatment. We did not account for any costs for those Chagas cases that did not complete treatment as treatment discontinuation increases with age, is more frequent in chronic disease patients, and tends to be better tolerated by younger patients.[14, 57] Also, the reasons for discontinuing treatment vary, making it difficult to determine the associated costs (e.g., rare or more severe side effects would lead to higher costs than treatment non-compliance). We assumed acute cases did not seek care and did not include these costs, while some cases are more severe and life-threatening (estimated to occur in 1–5% of patients), the vast majority are minor and unrecognized.[4, 58] Additionally, we did not include the cost of routine care and monitoring of indeterminate cases which may occur. However, our cost-benefit analysis shows that economic returns are garnered even with a high treatment cost per person, which could factor in these other costs.

### Conclusions

Treating Chagas cases in the acute and indeterminate stages, reduces transmission and new chronic cases (up to 18.0% and 68.2% relative reduction in transmission events and chronic cases in the Yucatán, respectively), provides health benefits, and would result in cost-savings within a few years, even when identifying and treating as little as 5% of cases annually. This supports the need for improved diagnostics and access to safe and effective treatment for earlier treatment of Chagas disease.
